# A Multidisciplinary Approach to High Throughput Nuclear Magnetic Resonance Spectroscopy

**DOI:** 10.3390/s16060850

**Published:** 2016-06-09

**Authors:** Hossein Pourmodheji, Ebrahim Ghafar-Zadeh, Sebastian Magierowski

**Affiliations:** Department of Electrical Engineering and Computer Science, Lassonde School of Engineering, York University, Toronto, ON M3J1P3, Canada; pmodheji@cse.yorku.ca (H.P.); magiero@cse.yorku.ca (S.M.)

**Keywords:** CMOS, low noise amplifier (LNA), Nuclear Magnetic Resonance

## Abstract

Nuclear Magnetic Resonance (NMR) is a non-contact, powerful structure-elucidation technique for biochemical analysis. NMR spectroscopy is used extensively in a variety of life science applications including drug discovery. However, existing NMR technology is limited in that it cannot run a large number of experiments simultaneously in one unit. Recent advances in micro-fabrication technologies have attracted the attention of researchers to overcome these limitations and significantly accelerate the drug discovery process by developing the next generation of high-throughput NMR spectrometers using Complementary Metal Oxide Semiconductor (CMOS). In this paper, we examine this paradigm shift and explore new design strategies for the development of the next generation of high-throughput NMR spectrometers using CMOS technology. A CMOS NMR system consists of an array of high sensitivity micro-coils integrated with interfacing radio-frequency circuits on the same chip. Herein, we first discuss the key challenges and recent advances in the field of CMOS NMR technology, and then a new design strategy is put forward for the design and implementation of highly sensitive and high-throughput CMOS NMR spectrometers. We thereafter discuss the functionality and applicability of the proposed techniques by demonstrating the results. For microelectronic researchers starting to work in the field of CMOS NMR technology, this paper serves as a tutorial with comprehensive review of state-of-the-art technologies and their performance levels. Based on these levels, the CMOS NMR approach offers unique advantages for high resolution, time-sensitive and high-throughput bimolecular analysis required in a variety of life science applications including drug discovery.

## 1. Introduction

Drug discovery is a costly and time consuming undertaking. It takes years for a few potential drugs to make it to the market. The first phase of this endeavor starts with the identification of potent therapeutic molecules. The most appropriate among these molecules are retained for the next phases of the drug discovery process, typically consisting of validation, characterization of pharmacodynamics, and toxicology studies for selected drug candidates, which is followed by intensive clinical studies [1]. Nuclear Magnetic Resonance (NMR) Spectroscopy systems are used intensively throughout all the above mentioned phases, along with other ancillary spectroscopic and characterization techniques such as liquid chromatographs coupled to mass spectrometers (LCMS) and ultraviolet (UV) spectrometers [2,3,4]. The conventional NMR systems can non-invasively and in a label-free manner identify chemical species present within a given sample. However, these systems suffer from an inherent lack of sensitivity required for many bio-molecular studies. Despite great advances in drug discovery using NMR technologies, the challenge of high throughput NMR spectroscopy for accelerating the drug discovery process remains unmet.

**Figure 1:**
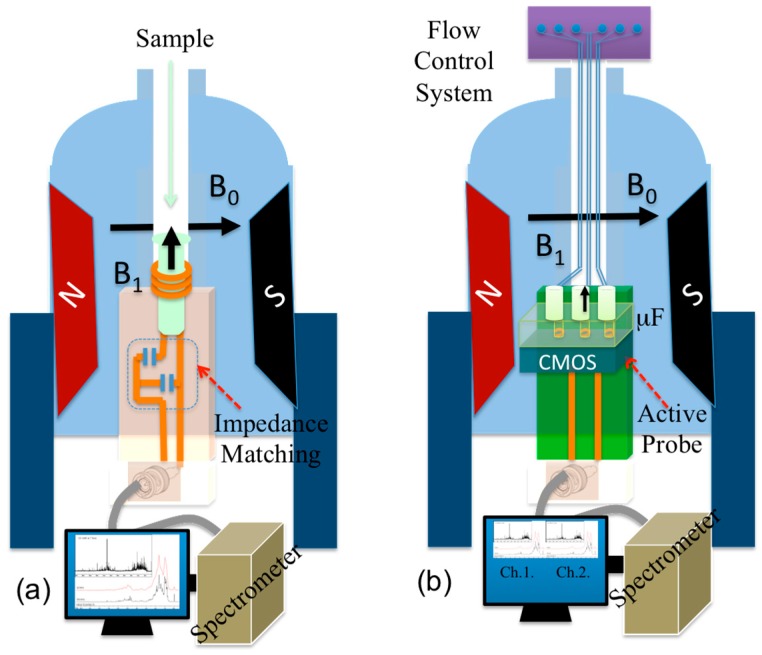
NMR Spectroscopy: Illustrations NMR system with (**a**) passive NMR and (**b**) active probe (μF = Microfluidic).

NMR is a phenomenon in which atomic nuclei absorb and re-emit a radio frequency (RF) electromagnetic signal in the presence of a uniform static magnetic field *B*_0_. The RF pulse is centered on a specific frequency to generate a magnetic field *B*_1_. This frequency *f*_0_ can be obtained from the following equation [5].

(1) $$f_{0}=\frac{\gamma B_{0}}{2\pi }$$

where the gyromagnetic ratio *γ* is a constant intrinsic to each nucleus, for the hydrogen proton ^1^H, *γ* = 42.6 MHz/T and for phosphorus ^31^P, *γ* = 13 MHz/T where T is the magnetic flux density unit (Tesla). After a certain time and due to magnetic induction, a RF signal is received through the coil. This signal contains information associated with the chemical composition of the molecular compound where the targeted material’s nuclei are located. A conventional NMR system, namely one-dimensional (1D) NMR employs a passive NMR probe as shown in Figure 1a. This probe consists of a RF coil surrounding the sample and a matching network between the coil and spectrometer. The goal of this work is the development of active NMR probe using CMOS technology as seen in Figure 1b. To date, various NMR technologies with different *B*_0_ ranging from low magnitudes on par with the earth’s magnetic field (*B*_0_ = 0.56 G, Terranova, Magritek Inc., Wellington, New Zealand) to the world’s largest superconducting magnetic field (*B*_0_ = 0.5 T, AVANCE 1000, Bruker Inc., Billerica, MA, USA) have been successfully commercialized for various life science applications.

**Figure 2:**
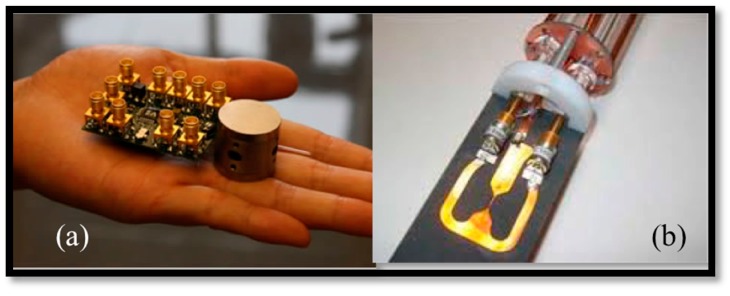
Emerging NMR technologies (**a**) CMOS based palm NMR [6] and (**b**) μSlot incorporated with NMR probe [7].

Furthermore, landmark research works have also been reported recently working toward the development of emerging NMR technologies [6,7]. Among these, Sun *et al.* reported the palm NMR system for Point-of-Care (PoC) diagnostics purposes (Figure 2a). Their proposed NMR probe consists of 2-mm diameter planer micro-coils (µCoils) integrated on a CMOS chip along with other conditioning circuitries [6]. This tiny electronic active probe is placed in a 0.5-T magnet (GMW Inc., San Carlos, CA, USA) for relaxometry analysis. In another effort, Maguire *et al*. demonstrated the great advantages of micro-slot (µSlot) structures incorporated with conventional NMR (see Figure 2b) for high-resolution NMR spectroscopy purposes [7]. In this direction, our team recently presented the design of an active CMOS active probe incorporated with a NMR probe for spectroscopy analysis in [8]. As a continuation of this work, in this paper, we discuss the challenge of developing an active NMR probe along with its associated design strategies. In the remainder of this paper, we discuss 1D and 2D NMR challenges, then we review the emerging NMR technologies in Section 3. The design strategies of an active NMR probe and simulation results will be discussed and demonstrated in Section 4 and Section 5. These sections will be followed by a conclusion in Section 6.

## 2. Related Works

In this section, we discuss the main challenges and related emerging technologies. The main challenges to the effective realization of high-throughput NMR spectroscopy center on the Signal to Noise Ratio (SNR). Herein, we also discuss the recent advances related to the development of µCoils and CMOS NMR techniques.

### 2.1. NMR Challenges

#### 2.1.1. SNR Challenge

The SNR of the spectrometer is described by Equation (2) [9].

(2) $$SNR=\frac{n\gamma^{2.5}\sqrt{tB_{0}^{3}}}{\sqrt{2T\left(R_{c}+R_{s}\right)}}$$

where *n* is the quantity of spins in the detector and *t* is the total experimental time, *γ* is the gyro-magnetic ratio of the nucleus, *R_S_* and *R_C_* are the resistances of the sample and the coil, and *T* is the temperature of the sample, coil and preamplifier. There is little opportunity to increase SNR through increases in *n* and *t*, especially at low concentrations, itself a desirable goal for miniaturized NMR scenarios. However, the SNR can be increased in different ways [10,11,12]. Given the scaling of SNR with (*B*_0_)^3/2^, (*T*)^−1/2^ in Equation (2) we see that increases in the spectrometer field strength and reduction in operating temperature lead to high sensitivity. The advent of high magnetic-field NMR systems and cryogenic probe technology (CryoProbe™, Bruker Inc., Billerica, MA, USA) have given a new means to study low concentrations of biomolecules through the generation of a higher NMR signal and the reduction of thermal noise, respectively. Using a cryogenic probe, the SNR has been increased fourfold over a conventional probe [13,14]. At this point (high *B*_0_ and low *T*), sensitivity is limited by RF power dissipation within the sample as encapsulated by *R_S_* [15,16]. In this case, the miniaturization of RF coils and consequently the miniaturization of the sample is a viable solution for increasing the unit-mass sensitivity of NMR experiments. The micrometer range sample holders minimize *R_S_* so that higher SNRs could be obtained and so that heating of samples containing thermo-sensitive molecules (e.g., proteins) could be avoided. Therefore, the downsizing of NMR samples while maintaining the application of large *B*_0_ (~1.5 T) paves the way for the introduction of novel NMR probes with smaller µCoils instead of conventional large coils.

#### 2.1.2. Towards High Throughput Spectroscopy

An NMR spectrometer is the major piece of equipment in the bio-molecular analysis arsenal, an indispensable tool for pharmaceutical companies in their drug discovery research. After synthesis of a drug molecule for example, it is imperative to derive its molecular conformation. Visualization of molecular structures and their fast time-scale dynamics can elucidate the stability of important molecular phenomena, notably the folding and miss-folding involved in docking potential drug molecules onto target biomolecules (e.g., proteins), which require efficient techniques to perform faithful 3D rendering of the complex (drug-target) and its long-term stability (dissociation energy). The current derivation process of 3D molecular conformations when using 2D NMR spectroscopy relies on the repetition of numerous 1D NMR radio frequency (RF) pulse transmission/acquisition sequences where the delay between excitation of nuclei and acquisition of their responses are incremented at each repetition. Besides lengthening the overall time of an experiment caused by the repeated 2D experiments conducted to improve the SNR, this incrementing procedure makes each test very sensitive to temporal instabilities and leads to large noise ridges along the indirect dimension [17]. Hence, a computationally driven classical implementation of 2D NMR spectroscopy on contemporary 1D NMR systems is unsuitable for the study of short time-scale phenomena; to yield near-atomic resolution in visualizing molecular structures and sub-microsecond resolutions in the study of their dynamics, technological advances are needed.

As classical NMR devices trade resolution for a gain of speed, many interesting molecular events and intermediate states are excluded from 2D NMR analysis. Reducing the acquisition time of 2D NMR spectra without accumulating these blind-spots is thus important [18]. One intuitive approach to accelerate 2D NMR techniques is under-sampling of the indirect time dimension. In practice, due to the elusiveness of the short-lived molecular states, applying Fast Fourier Transforms (FFT) to RF signals acquired with lower sampling rates defeats the purpose of elucidating the molecular dynamics involved; for example, in proteins miss-folding or their enzymatic degradation [19]. Numerous other approaches have been proposed to overcome these limitations in obtaining 2D NMR spectra on commercial NMR systems in reduced times without drawbacks or tradeoffs, but in vain [20]. A NMR system with a large number of µCoils enables the efficient execution of 2D NMR spectroscopy for derivation of 3D molecular structures.

### 2.2. Emerging NMR Technologies

The development of the NMR-mouse™ by Magritek Inc., in 2004, was the first step towards miniaturizing NMR systems [21]. Its major innovation was the ability to perform NMR relaxometry using planar *µCoils* and solid-state magnets in inhomogeneous magnetic fields that are not otherwise appropriate for NMR spectroscopy. Recently, Bruker Inc. launched an NMR system called Coil-on-a-Chip™, which is now being commercialized [22]. The main application of these two products is MR imaging and not NMR spectroscopy. Indeed, NMR spectroscopy requires vertical µCoils for RF excitation and magnetic resonance recording with high magnetic field homogeneity (*B*_1_) that planar coils with comparable sizes of samples lack. Despite the above discussed disadvantage of planar micro-coil, as reported by Fratila *et al.*, the planar coil might be used for semi-NMR spectroscopy if the coil size and consequently sample is enough (larger than micro-Liter (µL) and nano-Liter (nL) samples) [23]. As described in the next subsections, many efforts have been made to develop such µCoils using micro-fabrication techniques.

#### 2.2.1. NMR µCoils

**Figure 3:**
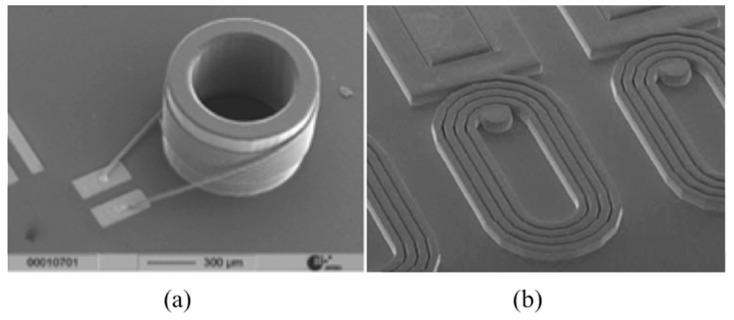
NMR μCoil techniques (**a**) Vertical μCoil [25] and (**b**) planer μCoil [27].

The advantage of using vertical *µCoils* instead of planar *µCoils* has been previously demonstrated in monitoring glioblastoma therapy by detecting micro-vesicles that have smaller sizes in comparison with tumor cells detected formerly by NMR systems with conventional planar coils [24]. To date, several efforts have been made to develop on-chip vertical *µCoils* using various micro-fabrication and rapid prototyping techniques. For instance, wire-bonding techniques have recently been used to develop an off-chip array of *µCoils* (see Figure 3a) by winding a 25-μm thick gold wire around micro-fabricated pillars (150–600 μm) [25]. Such RF *µCoils* (*Q* > 50 at *f*_0_ = 400 MHz) are good candidates for capillary NMR analysis. As a follow-up to this approach, Badilita *et al.* have recently reported the use of a wire-bonding technique in developing a micro-scale MRI solenoid incorporated with Lab-on-Chips [26].

#### 2.2.2. Integrated CMOS NMR Probe

The great success of RF CMOS technology for various mobile applications has inspired the idea of using this IC platform technology for NMR applications by developing an integrated NMR µCoil and their related circuits on the same chip. RF CMOS techniques can efficiently be employed to develop NMR spectrometers as reported by a number of researchers. Among these researchers, Cherifi *et al.* reported active matching circuitry integrated with a planar µCoil (see Figure 3b) for NMR applications using a 2T static magnet [27]. This active device prevents the injection of further noise due to passive impedance matching (see Section 4).

In 2008, another research group from Harvard University successfully used planar millimeter-sized coils to perform rapid analysis of biomarkers in fluid samples [6]. Likely because of the weak filling factor and the non-uniformity of the magnetic field generated by the planar *µCoils*, magnetic nanoparticles were used to concentrate the target-analytes at the coils’ centers for an increased sensitivity. Despite the great advantages of innovative nanoparticle-based, hand-held NMR relaxometry for diagnostic purposes, the use of magnetic nanoparticles prevents this technique from being used for analyzing molecular dynamics through spectroscopy as the presence of nanoparticles around macromolecules alters their interaction dynamics, and the relaxation times of nuclei become dispersed when the nanoparticles are magnetic in nature [28]. Indeed, molecular analysis for drug discovery requires a NMR *µCoil* with high filling factors—the volume of the sample is as large as the surrounding coil—in the absence of any magnetic labels. It is noteworthy that the planar µCoil is not an ideal solution for the challenge of high throughput CMOS NMR spectroscopy due to its non-homogeneity and the required large silicon area. Therefore, undeniably, the development of the vertical μCoil is the key step towards the development of NMR spectroscopy.

## 3. Design Strategy

This section first describes the main criteria to design active NMR probes. Then, we put forward the design of the NMR system featuring µCoil arrays and associated circuitries.

### 3.1. Design Criteria

The main design criteria to develop high-throughput, high resolution and programmable NMR probe for drug discovery and other high precision bio-molecular analysis are as follows.

#### 3.1.1. High Static Magnetic Field *B*_0_

The key challenge for high precision NMR spectroscopy is the development of larger magnets with higher *B*_0_. This is because the higher the *B*_0_, the higher the SNR that can be achieved (see Section 2.1). On the other hand, the miniaturization of coils surrounding the sample is an approach toward the development of high resolution NMR spectroscopy. The miniaturization of NMR coils results in lower *R_S_* and *R_C_* (see Equation (2)) and consequently increases the SNR represented by Equation (2). Therefore, a miniaturized NMR probe placed in a high magnetic field is the best candidate for enabling high-precision NMR spectroscopy. An array of µCoils can be incorporated with a miniaturized NMR probe using a standard microelectronic technology such as CMOS. A NMR CMOS chip with a large number µCoils can be placed inside of high static magnetic field for high throughput NMR spectroscopy purposes. It is noteworthy that the miniaturization of the NMR static magnet and associated circuits (see Section 1) [6] is another approach for the development of portable NMR based sensors for PoC disease diagnostic applications, however, the development of such portable NME systems is not the focus of this paper.

#### 3.1.2. Homogeneity

*µCoils* are the heart of miniaturized NMR systems. The uniformity of the RF magnetic field *B*_1_ generated by these µCoils during the excitation period is the key-challenging factor to achieve high resolution NMR spectroscopy. However, in practice, the uniformity of the magnetic field generated by µCoils/mini-coils can only be suitably achieved in the center of coils. For this reason, in such µCoils, *µL* and/or nL sample should be delivered to the center of µ-mini-Coils using time-sensitive and highly accurate µF (microfluidics) techniques. In the next section, we discuss the advantage of vertical µCoil for generating a homogenous *B*_1_.

#### 3.1.3. Ultra-Low Noise Voltage Amplifier

According to the Bloch decay equation [29], the NMR signal is in proportion to the equivalent macroscopic magnetic moments, which are proportional to the total number of nuclei. Therefore, µCoils surrounding *µL/nL* samples can generate small NMR signals. This small signal should be boosted with a very low noise amplifier (LNA) prior to its output connection via a coaxial cable; the LNA/coax interface needs to be made via an appropriate impedance matching circuit. Indeed, conventional passive NMR probes include an impedance matching system to assure the power loss is minimized when the signal is transmitted to the spectrometer. Since the impedance of µCoils is very much lower than conventional ones, the passive impedance matching may in fact further attenuate the signal unless it possesses very little loss. Therefore, the design of an active impedance matching circuit instead of a passive one is crucial as discussed in [28]. In other words, the electronic circuitry should be incorporated along with the NMR probe and placed inside the NMR system with high static magnetic field *B*_0_. As discussed in the next section, thanks to the high input impedance of the LNA, the design and implementation of a noiseless passive voltage pre-amplification prior to a high voltage gain LNA is the best solution.

#### 3.1.4. NMR Compatible Active Probe

The active NMR probe is a new approach relying on existing NMR technology with large static magnetic fields. This is why the compatibility of active NMR and standard NMR technologies is crucial. As already mentioned, a conventional NMR probe includes a coil connected to spectrometer through a passive impedance matching circuitry. Therefore, the spectrometer can transmit the RF pulse or record the magnetic resonance signal from this probe in both directions. An active probe including a large number of µCoils and LNAs cannot be used as dual path probe similar to a passive NMR probe. In other words, the design of an active probe compatible with standard NMR systems requires the development of a smart control system.

**Figure 4:**
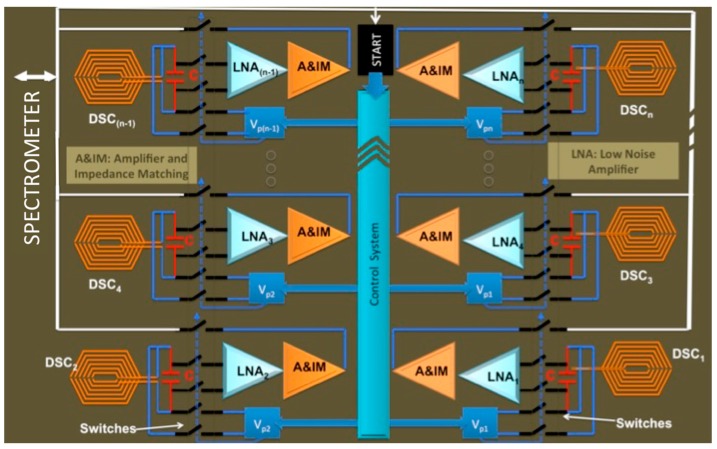
High throughput CMOS based Active NMR.

As seen in Figure 4, a high throughput active probe consists of an array of µCoils and their associated pre-amplifiers, LNAs, and impedance matching active circuitry. The digital system consists of programmable pulse generators (V_p1_, V_p2_ and *etc.*), multiplexer and associated switches to select each µCoil individually. This smart digital system detects the signal sent by the spectrometer and starts NMR spectroscopy with a new protocol. Additionally, this smart system is also important to control the timing and analog signal transmission toward the external spectrometer. The core of this design, the µCoil and amplifier are discussed in Section 3.2 and Section 3.3.

### 3.2. µCoils

**Figure 5:**
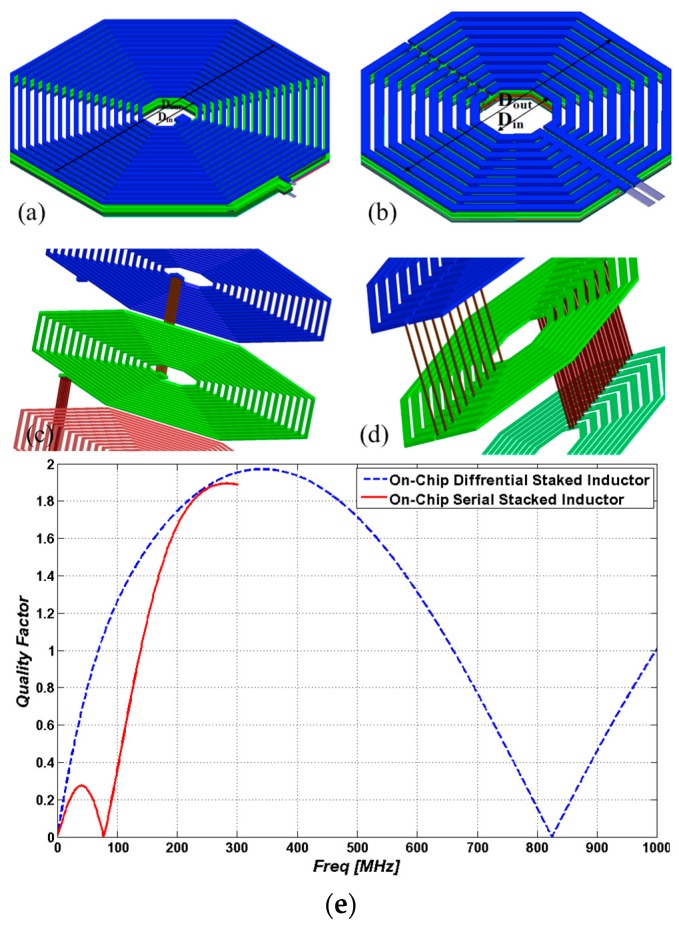
Magnetic field simulation results: (**a**,**c**) SSC; (**b**,**d**) DDS and (**e**) quality factor of SSC and DSC.

CMOS technology, by offering multiple metal layers, is the best candidate to design three dimensional (3D) µCoils with specific geometry suitable for high resolution NMR spectroscopy. In this section, we demonstrate and discuss the design and simulation results of two different µCoil structures, namely serial stacked coils (SSC) and differential stacked coils (DSC) shown in Figure 5a,b.

#### 3.2.1. Serial Stacked Coil (SSC) *vs.* Differential Stacked Coil (DSC)

SSC is a simple structure consisting of multiple planar coils in different layers (with *i* layers). These planar coils (with *j* turns) are connected through inter-metal vias in order to achieve large inductance values as seen in Figure 5c. However, the equivalent resistance will be increased as expressed in Equation (3) [30].

(3) $$R_{eq-SSC}=\underset{i}{\sum }\underset{j}{\sum }R_{i,j}$$

The DSC is a symmetrical differential structure consisting of multiple planar µCoils (*i* layers, and *j* turns in each layer) that are connected to each other with two vias [31,32]. The equivalent resistance of a DSC structure as expressed in Equation (4)

(4) $$R_{eq-DSC}=\underset{i}{\sum }{\left(\underset{j}{\sum }R_{i,j}{}^{-1}\right)}^{-1}$$

As described in [8], in our design, each layer consists of multiple parallel coils in order to increase the magnetic field *B*_1_. Therefore, a number of vias are used to connect the µCoils between two layers as seen in Figure 5d.

The equivalent circuit model of both DSC ad SSC structures is a tank circuit and therefore the quality factor of their equivalent circuits can be expressed by

(5) $$Q_{L\left(\omega \right)}=\frac{\omega L_{eq}}{R_{eq}}\left(1-\frac{C_{eq}R_{eq}^{2}}{L_{eq}}-\omega^{2}L_{eq}C_{eq}\right)$$

where *L_eq_* and *C_eq_* are the equivalent inductance and capacitance and *R_eq_* is the equivalent resistance that can be obtained from Equation (3) or Equation (4) for EEC and DSC structures, respectively. Based on Equations (3)–(5), one can roughly conclude that quality factor of the DSC structure is higher than the SSC structure. This is because *R_eq_*_-SSC_ >> *R_eq_*_-DSC_. Next, in Section 3.2.2, we calculate the quality factor using the finite-element electromagnetic simulator HFSS.

#### 3.2.2. Geometry Design, Modeling and Optimization

Two different topologies of the stacked inductors (DSC and SSC) are designed in a CMOS compatible manner using eight metal layers. In this design, from a circuit point of view, two parameters are important for designing µCoils. These parameters are the self-resonant frequency *f*_0_ and the quality factor, which determines the noise level of the µCoil. Additionally, in the design of µCoils, the silicon area is another very important factor that should be minimized. The ANSYS HFSS software is employed to search for an optimum geometry at *f*_0_ = 300 MHz. Based on these studies, the quality factors of both structures *versus* frequency are obtained as shown in Figure 5e.

The optimum inner and outer diameters of the hexagonally shaped DSC structure are 80 and 365 µm respectively. The same geometry was also used for the SSC structure. As the results of HFSS modeling and simulation show, the operating frequencies of SSC and DSC structures are 40 MHz and 300 MHz, respectively as seen in Figure 5e. As expected (see Section 3.2.1) due to the differential configuration of DSC, the quality factor of the differential inductor is around 2, a value seven times better than the SSC structure’s quality factor.

#### 3.2.3. Homogenous Magnetic Field

**Figure 6:**
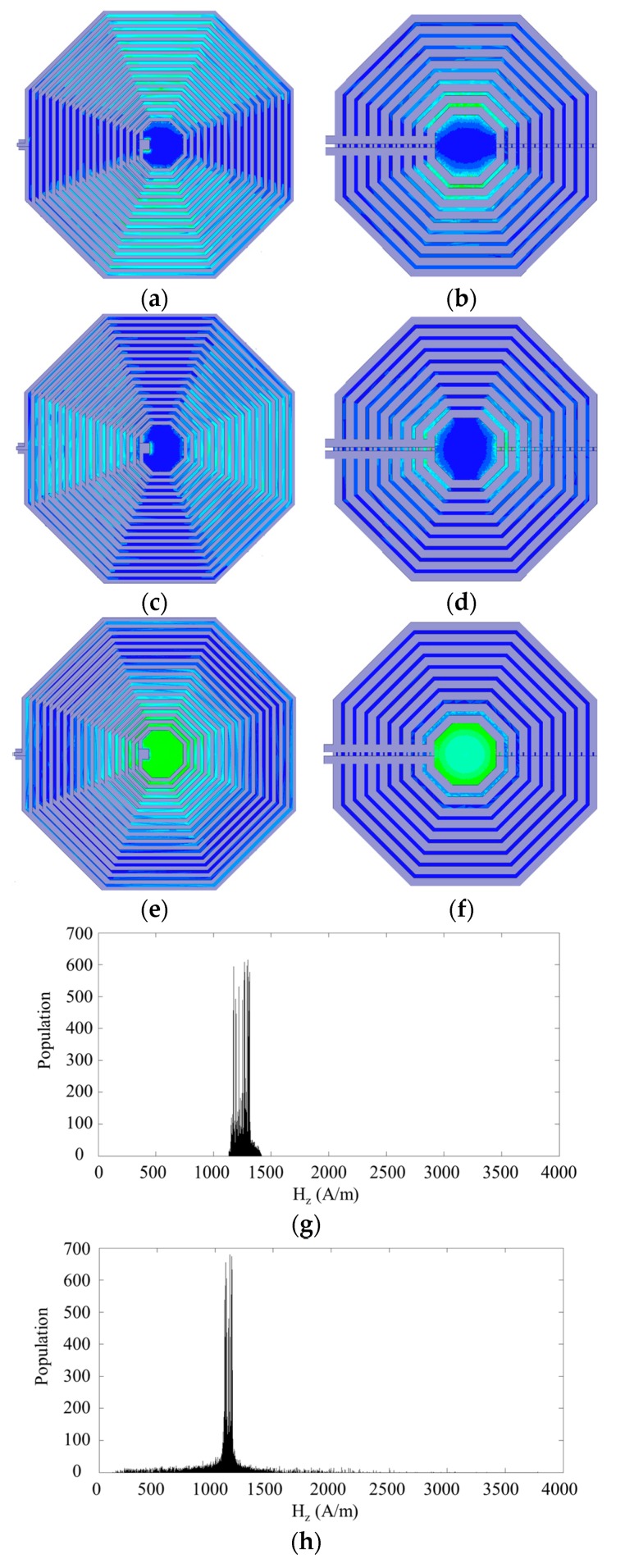
Magnetic field (*B*_1_) simulation results: (**a**,**c**,**e**) *B*_1*x*_, *B*_1*y*_, *B*_1*z*_, generated by SSC, respectively; (**b**,**d**) and (**f**) *B*_1*x*_, *B*_1*y*_, and *B*_1*z*_, generated by DSC respectively; uniformity of magnetic field in the (**g**) DSC and (**h**) SSC models. Population defines as the number of pixels in the center of micro-coils with the same magnetic field strength (Hz).

Another advantage of the DSC structure is the homogeneity of the magnetic field *B*_1_ generated inside the µCoil. As seen in both structures (see Figure 5c,d), the metal vias introduce horizontal magnetic field components which affect the homogeneity of the magnetic field *B*_1_. In the DSC structure, the current flows through the vias between each two layers are in opposite directions. These opposite current directions almost cancel the horizontal magnetic field components. Therefore, one can argue that the symmetric structure of the DSC can almost cancel out the magnetic parasitic effects while the asymmetric structure of the SSC cannot. The magnetic field generated by these asymmetric and symmetric structures in three different directions (*B*_1*x*_, *B*_1*y*_ and *B*_1*z*_) is demonstrated in Figure 6a–f.

As seen in these figures, the magnetic fields in the *x* and *y* directions are less than the magnetic field in the *z* direction in both DSC and SSC structures in the middle of the coils. The ratio of the *B*_1*xy*_ over *B*_1*z*_ is 3.02% (33.67/1114) at the center point of the SSC while this ratio is 0.93% (11.89/1274) at the center point of the DSC. Based on these values, the vertical magnetic field *B*_1*z*_ in both structures are close to each other while the DSC has lower magnetic field in the *xy*-plane in comparison to the SSC structure. In addition, one can see the distribution of the *B*_1*z*_ in the middle volume of the DSC and SSC structures, in Figure 6g,h, respectively. Based on these results, the mean values of the *B*_1*z*_ for the DSC and SSC are 1290 A/m and 1115 A/m, respectively. Furthermore, the standard deviation of the DSC is around 59.5 A/m which is about half of the standard deviation of the SSC which is about 108 A/m. Based on all these results, we can say that the uniformity of the magnetic field in the DSC structure is much better than that of the asymmetric structure. This is the key advantage of the DSC structure for NMR spectroscopy. The computational results shown in Figure 6g,h prove this concept. In these figures the population *vs.* magnetic field strength are shown. The central cross sections each coil is devided into a large number of small square or so-called pixel. Total population of pixels with certain magnetic stregnth *H_z_* is ploted in Figure 6g,h. Therefore, thess plots can show the uniformity of magnetic field in the center of the microcoil with a DSC structure is much better the one with a SSC structure.

It is noteworthy that the quality factor of μCoils implemented in CMOS is lower than the quality factor of μCoils reported in [25]. This low quality factor quality is due to the thin underneath aluminum layers in CMOS technology. On the other hand, CMOS offers the advantage of developing multiple μCoils along with conditioning circuitries on a single chip. Herein, we improved the quality factor by connecting multiple layers using vias between them. The winding density and diameter of μCoils shown in Figure 6 are about 17 turns/360 micron.

### 3.3. Low Noise Amplifier (LNA)

As described in Section 2.1, the design of the low noise amplifier is key for the development of high resolution NMR spectroscopy. A low noise amplifier system consists of two parts: a pre- and a post-amplifier.

#### 3.3.1. Front-End Pre-amplifier

**Figure 7:**
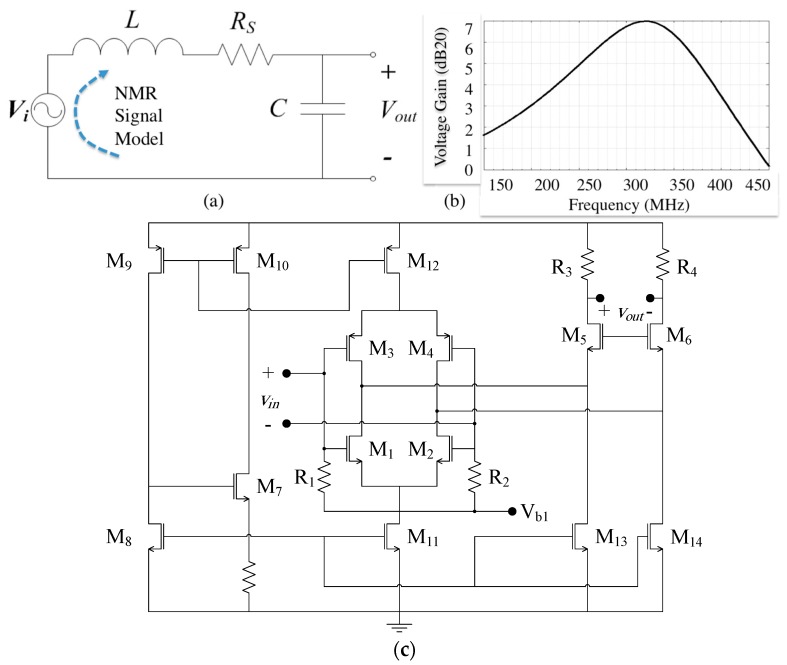
Low noise amplifier (**a**) Pre-amplifier equivalent circuit (**b**) voltage gain of preamplifier; (**c**) schematic of LNA circuitry.

As already mentioned in Section 4.1, it is very crucial to replace the impedance matching with a passive voltage amplifier as reported in [6,32]. Figure 7a shows the circuit model of such a passive pre-voltage amplifier. This LC resonator consists of an on-chip DSC inductor in parallel with a tuning capacitor. A MIM capacitor in a CMOS process is used for this purpose. By assuming that LNA’s input impedance is very high, the voltage gain of this pre-amplifier can be obtained from the following equation.

(6) $$\left|A_{v}\right|=\frac{1}{\sqrt{{\left(1-LC\omega^{2}\right)}^{2}+{\left(R_{s}C\omega \right)}^{2}}}$$

The optimum capacitance can also be obtained by taking differential of Equation (6) at *C* ((δAv)/δC=0))

(7) $$C=\frac{L}{{\left(L\omega \right)}^{2}+R_{s}^{2}}$$

Therefore, by choosing *C* appropriately, the voltage gain peaks as shown in Figure 7b, at a frequency of around 300 MHz. Indeed, this input LC structure (pre-amplifier) boosts the weak NMR signal with minimal noise corruption for the ensuing signal processing. This pre-amplification is followed by a post amplifier shown in Figure 7c.

#### 3.3.2. Post Amplifier

The post-amplifier is a fully differential cascode push-pull LNA that is designed and implemented for NMR spectroscopy purposes [8]. In this circuit, *M_1_–M_4_* form the core of the LNA and constitute a differential cascade voltage amplifier circuit to suppress the common mode noise. In this sub-circuit, M_1_–M_2_ are NMOS and M_3_–M_4_ are PMOS transistors. The total voltage gain can be obtained from.

(8) $$\left|A_{v-LNA}\right|=\left(g_{m1}+g_{m3}\right)R_{3}$$

where *g_m_*_1_ and *g_m_*_3_ are the transconductances of the NMOS and PMOS input transistors and *R_3_ = R_4_* are output resistive loads. *M_7–12_* and *R_1_ = R_2_* constitutes a bootstrap voltage reference’s circuitry to regulate bias of amplifier. This circuitry is used to minimize the thermal effect on biasing of circuitry. On the other hand, *M_13_–M_14_* as well as *M_11_* mirrors the bias currents of M_8_.

For noise performance, we need to optimize the noise source of *M_1_–M_4_*. According to the circuit shown in in Figure 7c, the input referred noise due to M_1_ and M_3_ can be stated as follows.

(9) $$\bar{V_{n,\;in}^{2}}=8kT\gamma \left(\frac{1}{g_{m1}+g_{m3}}\right)+4kT\left(\frac{1}{{\left(g_{m1}+g_{m3}\right)}^{2}R_{3}}\;\right)\;$$

where *T*, *k* and *γ* are temperature in Kelvin, Boltzmann constant and the drain thermal noise excess factor respectively. Based on this equation, the noise can be reduced by increasing the DC currents or *g_m_*_1_ and *g_m_*_3_. However the extra DC current increases the power consumption and consequently the temperature that results in higher thermal noise level. In other words, by designing low-power-consumption circuitry, we can prevent the self-heating of the chip [33].

## 4. Results

### 4.1. LNA Post-Layout Simulation

**Figure 8:**
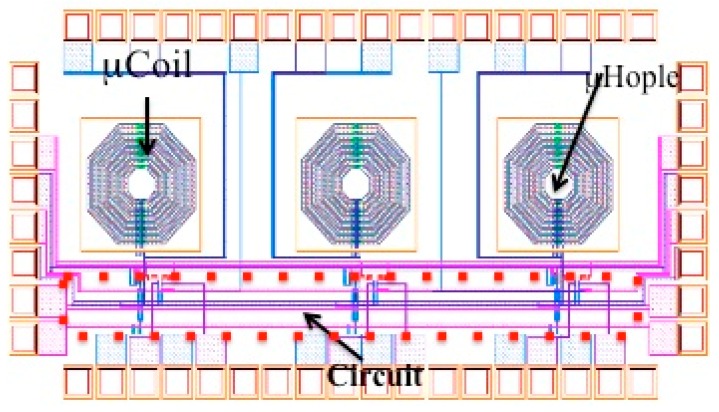
CMOS layout revealing three moils and associated pre- and post-amplifiers.

The layout and simulation results of the proposed integrated circuit are demonstrated and discussed in this section. The HFSS and Cadence design suites were used to model and simulate the µCoil and IC circuit, respectively. The layout of this circuit can be seen in Figure 8.

**Figure 9:**
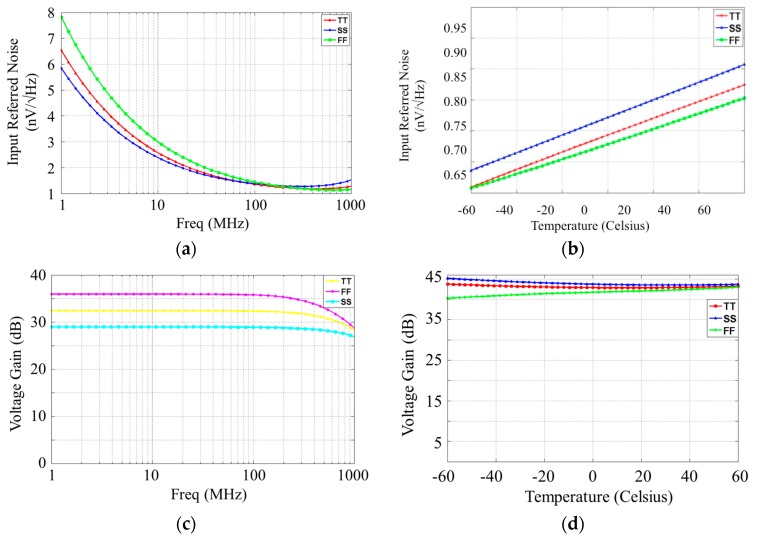
Simulated input referred noise of the (**a**) LNA over frequency and (**b**) the LC-LNA over temperature at 300 MHz for three fabrication process corners; Simulated voltage gain of the (**c**) LNA over frequency and (**d**) the LC-LNA over temperature at 300 MHz for three fabrication process corners.

As noted above, the LNA is designed to achieve an optimum input referred noise. Therefore, we designed the LNA with a large transconductance for input transistors to achieve about 780 pV/√Hz at 300 MHz. Figure 9a,b show the input referred noise of the LNA *versus* a range of frequencies and a range of temperatures respectively for three fabrication process corners.

Another important parameter of the LNA is the voltage gain. The voltage gain of the front-end receiver is around 43 dB at 300 MHz and this voltage gain for three fabrication process corners is shown in Figure 9b. The effect of temperature on voltage amplifier’s gain is also shown in Figure 9d. Some characteristics of the front-end receiver are summarized in Table 1. Since we impose the sample over the chip, the characteristics of the chip should not change to affect the functionality of the circuit. Therefore, both input referred noise and voltage gain of the front-end receiver are simulated for temperature variation (−60 °C to +60 °C) and shown in Figure 9c,d for three fabrication process corners, respectively.

### 4.2. Spectral Analysis

#### 4.2.1. Simulation Model

**Figure 10:**
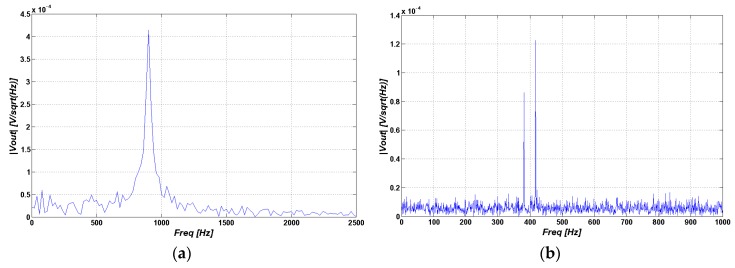

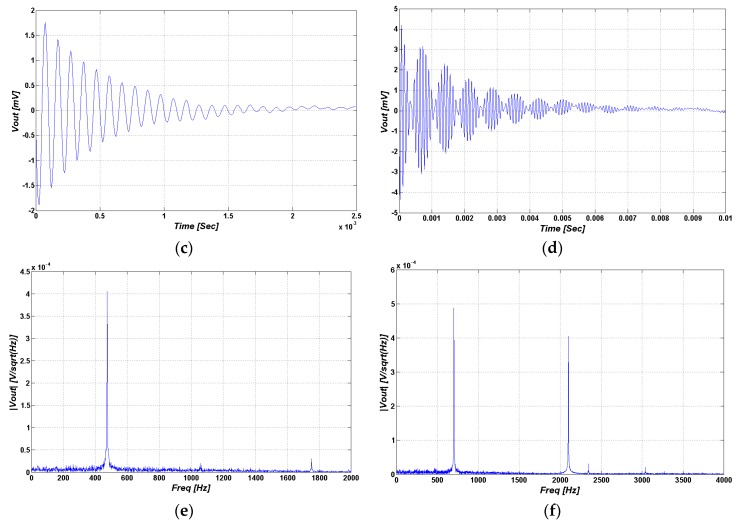
Simulated NMR spectrum for (**a**) -CH3 group (**b**) Creatine group, Simulated NMR signal of Water and Toluene (**c**,**d**) in time domain (**e**,**f**) frequency domain.

According to the Bloch equations [29], the NMR signal should conform to the relationship below:

(10) $$S\left(t\right)=S\left(0\right)e^{\left(1-\sigma \right)\omega_{0}j}e^{-t/T_{2}}$$

(11) $$S\left(0\right)\propto M_{eq}$$

where *ω*_0_ is the angular resonance frequency, *σ* is the isotropic nuclear shielding, and *T*_2_ is the spin-spin relaxation time. In this part, two NMR signals are modeled for the -CH3 group of Lactate and Creatine (Cr). For the first one the chemical shifts are 1.27 and 1.39 ppm and for the latter one it is 3 ppm; related to the reference frequency of 300 MHz. *T*_2_ is almost 1040 ms and 100 ms, respectively. By using these values, we can model the NMR signal, which we use as the input of our LNA. If we consider that bandwidth of the signal is 1.1 kHz for noise calculation, and the parasitic resistance of the coil is 10 Ω, then the resulting output spectra of all signals relative to the reference frequency of 300 MHz are shown in Figure 10a–f. The simulation results for water and toluene in the time domain and frequency domain are shown, as well. According to [11], water (H_2_O) has one chemical shift (*δ*) of 1.588 ppm and toluene (C_7_H_8_) has two chemical shifts of 2.34 ppm and 7 ppm. If we use Equations (10) and (11), we should find the relative isotropic nuclear shielding from associated chemical shift.

(12) $$\sigma =\frac{\sigma_{ref+\sigma_{sample}}}{1-\sigma_{ref}}$$

According to Equation (12), if we consider tetramethylsilane (TMS) as the reference whose chemical shift and isotropic nuclear shielding are zero, then we can derive the *σ* from the specific chemical shift. By this and (Equations (10) and (11)), the output signal of our circuit for water and toluene in both time domain and frequency domain can be obtained as shown in Figure 10. Figure 10a,b,e,f show the spectrum of output signal associated with various chemical materials. As demonstrated and discussed in the Section 4.2.2, these spectrums are very similar magnetic resonance spectrum of the corresponding chemical materials using a standard NMR system. For better comparison and understanding, we present an experimental platform and demonstrate the experimental results as detailed in Section 4.2.2.

#### 4.2.2. Experimental Model

**Figure 11:**
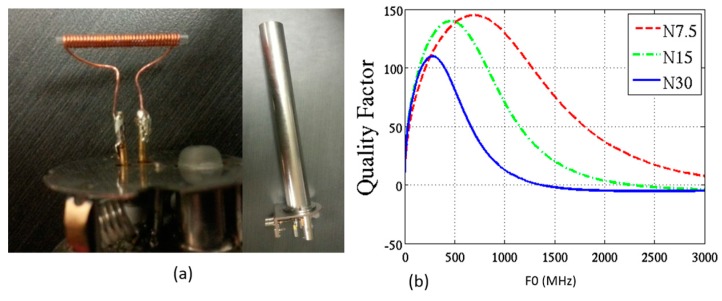
Experimental NMR (**a**) mini-coil connected to probe and a probe (300 MHz, Bruker) and (**b**) quality factor simulation results of mini-coil with different number of turns.

**Figure 12:**
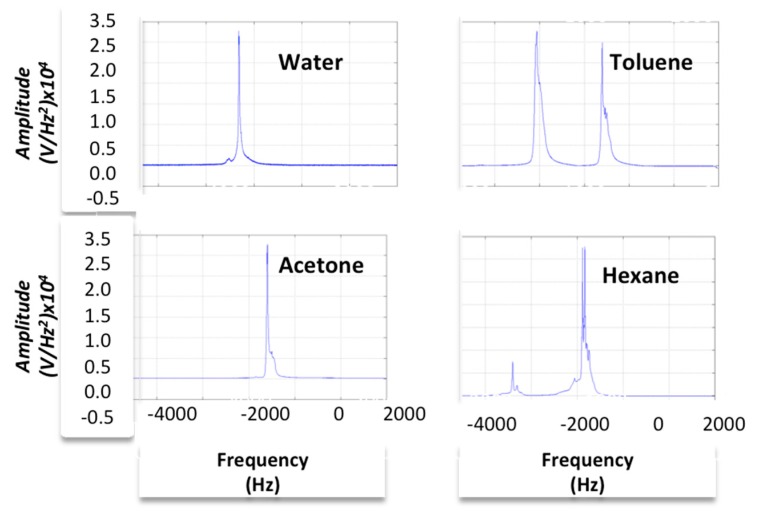
Mini-Coil Experimental results, Water, Toluene, Acetone and Hexane.

The above discussed spectral analysis is based on information documented in the literature. In this work, in order to verify the funcationality of proposed active probe and develop a more accurate model of the NMR resonance signal, we can perform the NMR experiment using the same chemical solutions. In this work, a mini-coil is designed, implemented and incorporated with a 300-MHz, Bruker NMR probe. This coil is used as reference to perform the experiments and compare with the experimental/simulation results of active device in the future. Figure 11a shows the mini-coil realized by winding a wire around a glass. This microtube is also used as a sample holder for NMR spectroscopy. This mini-coil was matched to a 50-Ω coaxial cable connected to the spectrometer. A low complexity procedure was performed to match the impedance by changing the number of turns according to the siminmulation shown in Figure 11. These HFSS simulation results reveal quality factor properties. Thereafter, the experiments were perfromed on various chemical solutions as shown in Figure 12. As seen in these figures, the spectrum of water and toluene is very similar to the simulated ones. Additionaly, we tested the mini-Coil using acetone and hexane.

## 5. Discussion

The development of a microhole inside CMOS based vertical coils is key toward realization of active NMR probes for drug discovery applications. Throughout this paper, we discussed the vertical μCoil and LNA and, in this section, the key post-processing factor is discussed. Indeed, the functionality of vertical coil depends on the fact that the sample is placed inside the coil. For this, the creation of micro-holes is crucial in this technique. Additionally, the development of high-throughput NMR spectroscopy should also be interfaced with a microfluidic structure in order to introduce the chemical samples to the NMR sensors.

*Post-Processing*: The creation of micro-scale through-CMOS holes at the center of the vertical µcoils will be a technological leap that will bring us closer to the development of disposable µF devices and reusable CMOS 2D-NMR systems. As described by Uddin *et al.*, electron-beam (e-beam) lithography can efficiently be used to drill tiny holes (Diameter <10 nm) in a membrane created above the CMOS chip [34]. The creation of the membrane above the CMOS chip using post-CMOS µfabrication processes is a key step toward development of various Micro-Electro-Mechanical-Systems (MEMS) like; micro-channels, micro-hot-plates and micro-cantilevers as reported in the literature [35]. Deep Reactive Ion Etching (DRIE) is also an important post-CMOS processing technique that can accurately back-etch silicon wafers of integrated chips and could also be used for the creation of through-CMOS micro-holes using photolithography masking techniques. However, we can study the possibility of using e-beam and Focused Ion Beam (FIB) etching techniques to increase the precision of the hole-drilling process used. It is noteworthy that e-beam and other etching techniques based on ionic diffusion into the etched materials like the FIB etching technique could prove to be inappropriate for drilling through-wafer holes, when these high aspect-ratio channels are in proximity to active devices inside the CMOS chip. The reason for this is the significant charge injection into active zones causing damage to the doped silicon. The challenge is to control the etching process, bringing it to a halt in proximity of active areas below the surface of the CMOS chip, in order to preserve the integrity of µE devices. Despite recent progress in creating nano-holes (to be distinguished from through-CMOS holes) on top of CMOS chips, developing an array of such ducts in proximity to integrated sensors and circuitry still represents a challenging endeavor. One can develop an array of vertical RF µcoils with through-CMOS IC µholes to direct the sample inside the µCoils using µF structures.

*Microfluidics*: This downsizing of NMR samples is an important step toward the development of high throughput NMR spectroscopy including a large number of μCoils. Therefore, in this direction, micro-fluidic systems play an important role in the field of NMR. To date, many papers have reported the development of various microfluidic techniques incorporated with NMR probe for NMR spectroscopy purposes [36,37,38,39,40]. Among various standard and non-standard fabrication processes, photolithography, by offering high resolution (<1 micron), is widely used for the development of microfluidic structures with various sizes ranging from a few millimeters down to about 10 μm. The combination of microfluidic structures and NMR probes has been created to direct the samples toward the sensing sites. Despite these great advances, the design and implementation of microfluidic structures for high-throughput NMR spectroscopy remains unmet. In this direction, we will study the development of a large array of microfluidic structure incorporated with a CMOS chip as an important step towards the development of high-throughput CMOS NMR spectroscopy.

## 6. Conclusions

In this paper, we presented a new approach toward the development of high-throughput NMR spectroscopy using CMOS microelectronic technology. µCoils associated with RF interface circuitries were designed and simulated as the core part of the NMR system. We presented a fully integrated CMOS multi-turn differential stacked detection coil and front-end receiver for NMR applications. We also employed a LC resonator as a pre-amplification element followed by the desired LNA optimized for the lowest input referred noise to accommodate the weak NMR signal. The multi-turn differential stacked inductor is integrated into the CMOS chip with the LNA. The desired on-chip detection inductor is implemented by using eight layers of a 0.13-µm CMOS technology. Using all eight layers of the technology allows us to reduce the size of the inductor at its specific operating frequency. Furthermore, the differential topology of the stacked inductor improves its quality factor without requiring changes to the fabrication process. Furthermore, this paper outlined the key emerging NMR technologies as well as the NMR challenges faced and discussed promising design strategies to overcome these. Based on these discussions, the active NMR probe is the best candidate for developing high throughput NMR spectroscopy and for accelerating drug discovery research and pharmaceuticals

## Figures and Tables

**Table 1:** CMOS Chip Specifications.

Technology	CMOS 0.13 µm
Area (mm^2^)	1 × 2
Vdd (V)	1.6
DC current of the LNA core (mA)	3.4
LNA 3-dB BW (MHz)	800
Voltage Gain (dB) @ 300 MHz	42.85

## Abbreviations

The following abbreviations are used in this manuscript:

NMR	Nuclear Magnetic Resonance
CMOS	Complementary Metal Oxide Semiconductor
RF	Radio Frequency
LNA	Low Noise Amplifier
